# Superficial fungal infections in adults in Northern Finland between 2010 and 2021: A register‐based study

**DOI:** 10.1002/hsr2.70138

**Published:** 2024-10-10

**Authors:** Teemu Mäntyniemi, Suvi Säntti, Eetu Kiviniemi, Jari Jokelainen, Laura Huilaja, Suvi‐Päivikki Sinikumpu

**Affiliations:** ^1^ Faculty of Medicine University of Oulu Oulu Finland; ^2^ Faculty of Medicine, Northern Finland Birth Cohorts, Arctic Biobank, and Infrastructure for Population Studies University of Oulu Oulu Finland; ^3^ Department of Dermatology Oulu University Hospital Oulu Finland; ^4^ Research Unit of Clinical Medicine University of Oulu Oulu Finland

**Keywords:** comorbidities, diagnostic delay, epidemiology, patient profile, superficial fungal infection

## Abstract

**Background and Aims:**

Superficial fungal infections are common dermatological reasons to visit a doctor in primary care in Finland. However, their variable clinical picture and minor symptoms may lead to delayed diagnosis. We aimed to investigate the epidemiology and patient profile of fungal infections treated in secondary care over a decade.

**Methods:**

This is a retrospective study including adult patients with a fungal infection in the scalp, nails, or superficial skin diagnosed at the Oulu University Hospital, Finland between the years 2010 and 2021.

**Results:**

There were 573 patients with male predominance (57.6%). All studied fungal infections were more common in the oldest age group (>61 years). The number of fungal infections increased from the year 2017 onward. Only one‐third (37.7%) of the patients were referred to the dermatology clinic because of a suspected dermatophyte infection, and in 46.0% of cases, the diagnostic delay exceeded 6 months. The most common fungal infection was tinea pedis (*n* = 295, 51.5%) followed by tinea unguium (*n* = 275, 48.0%); as concomitant infection, they were present in 108 (18.8%) of all patients. The most common pathogen causing a fungal infection was *Trichophyton rubrum*.

**Conclusion:**

During the study period, the incidence of diagnosed superficial fungal skin infections increased. There was a remarkable diagnostic delay from the onset of symptoms to diagnosis in these most common dermatological conditions.

## Key points

- Superficial fungal infections are highly prevalent and they cause burden and decrease the quality of life.
- We found that the incidence of superficial fungal infections treated in secondary care was increasing.
- Fungal infections affect both genders, but more often males and especially those who are older.
- There was a remarkable diagnostic delay in these diseases.

## 1 INTRODUCTION

Superficial fungal infections are highly prevalent, affecting around 20%–25% of people worldwide.^1^, ^2^ Their prevalence is probably underestimated and has been increasing because of modern life, increased traveling and immigration.^3^ In Finland, fungal infections are one of the most frequent dermatological reasons to visit a doctor in primary care.^4^ Fungal infections cause burden and decrease the quality of life because of discomfort, pruritus, and social aspects.^5^

In adults, the most common fungal infections are tinea pedis and onychomycosis.^6^, ^7^, ^8^ Typical predisposing factors for fungal infections are male gender, age, immunocompromised status, obesity, and diabetes.^2^, ^8^, ^9^ The clinical presentation of fungal infection varies between anatomical locations of the infection, and fungal infection can thus be easily misdiagnosed for other skin diseases like eczemas.^10^ In addition, fungal infections can present almost symptomless^10^ which may cause delay of the treatment.

The pathogens causing superficial fungal infections are commonly called dermatophytes, which are divided into seven genera the three most common being *Epidermophyton, Microsporum*, and *Trichophyton (T.)*.^3^, ^11^ In recent decades, *Trichophyton rubrum* has been the predominant pathogen in Europe causing superficial fungal infections.^3^, ^11^ However, there has been an increasing frequency of recalcitrant infections caused especially by *Trichophyton indotineae*.^12^ Due to the difficulties in diagnostics, in most cases, diagnosis of a fungal infection is recommended to be confirmed with microscopy, fungal culture, and/or nucleic acid amplification test (NAAT) based methods.^11^, ^13^

There are only a few epidemiological studies about superficial fungal skin infections, especially ones performed in Europe.^6^, ^14^, ^15^ The aim of this study was to investigate the epidemiology and diagnostic delay of superficial fungal infections as well as the patient profile and comorbidities of affected adults treated in secondary care in Northern Finland between 2010 and 2021.

## 2 MATERIALS AND METHODS

**Figure 1:**
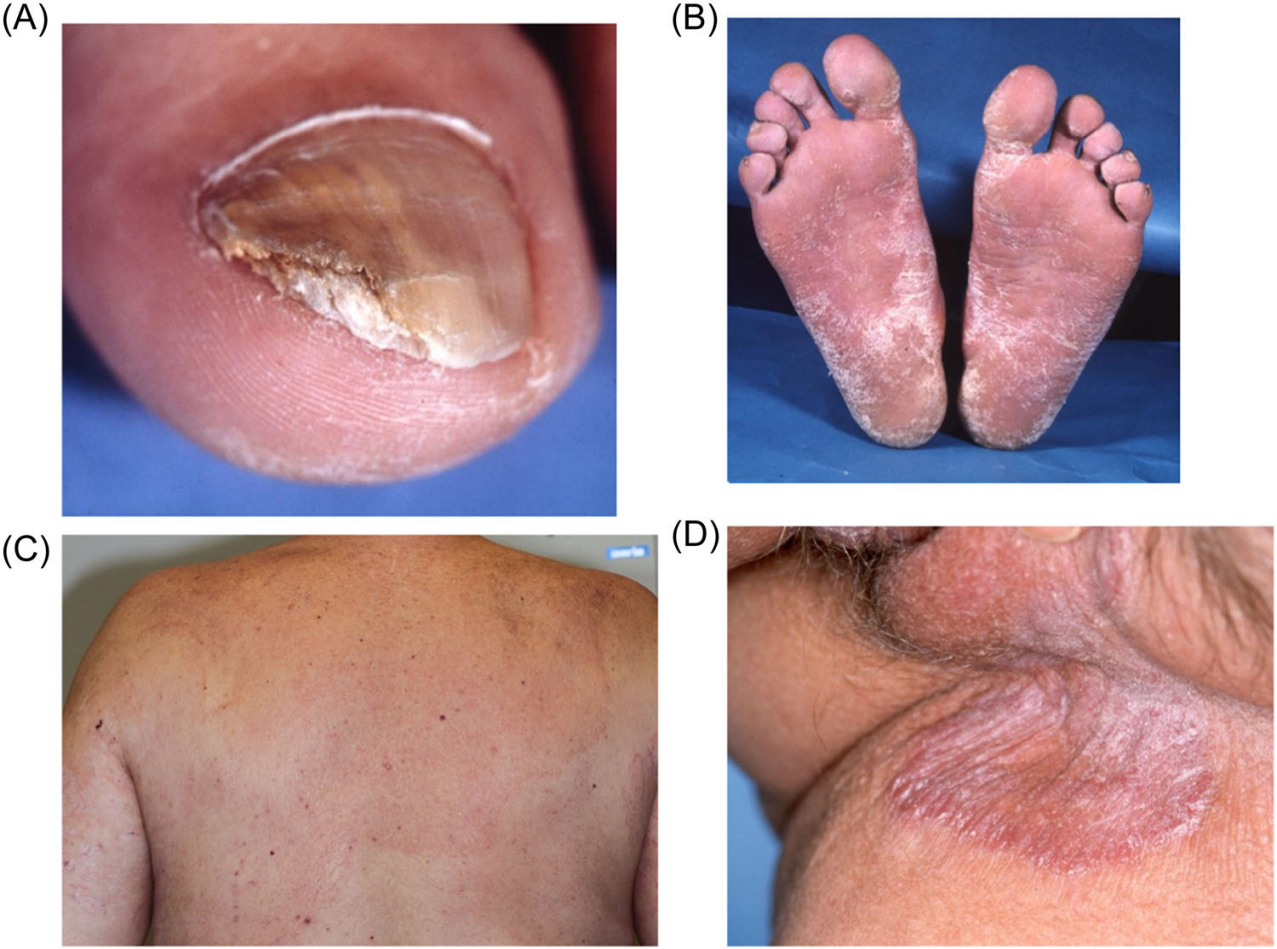
The clinical picture on (A) tinea unguium of toes, (B) tinea pedis, (C) tinea corporis, and (D) tinea cruris. Photos: Archives of the Department of Dermatology, Oulu University Hospital.

This study included all patients aged ≥18 years who visited the dermatology outpatient clinic of Oulu University Hospital (OUH) between 2010 and 2021 and were diagnosed with a superficial fungal infection. OUH is the only hospital with a department of dermatology in the Northern Ostrobothnia Hospital District (NOHD), providing secondary healthcare for about 413,000 people. Cases were identified from electronic health records (EHR) of OUH using the following codes based on the International Classification of Diseases (ICD‐10): B35.0 (tinea capitis), B35.1 (tinea unguium of toes or fingers), B35.3 (tinea pedis), B35.4 (tinea corporis), and B35.6 (tinea cruris) (Figure 1). The data from EHR was collected manually by T. M. and S. S., and only patients with a final diagnosis of fungal infection were included in the study (i.e., cases with false ICD coding were excluded). The included patients had their diagnosis verified with NAAT‐based methods (in more details, polymerase chain reaction [PCR]), fungal culture or skin biopsy, or had typical clinical presentation for superficial fungal infection in clinical evaluation by a dermatologist. PCR was carried out with DermaGenius® 2.0. complete multiplex kit (PathoNostics) which was run on the Rotor‐Gene Q (Qiagen) according to the manufacturer's instructions. Culture of samples was performed in accredited clinical mycology laboratories (Finland). Fungal identification was based on the macromorphological characteristics of fungal colonies and their micromorphological characteristics.

Data from EHR was collected into an electronic data collection tool (REDCap) and included following items: age, gender, the year of diagnose, whether the fungal infection was the reason for referral from primary care, the diagnostic delay from the onset of the symptoms, the history of previous fungal infections, the location of the fungal infection, the diagnostic method, the genera of the pathogen, and the presence of comorbidities (diabetes, obesity, psychiatric diagnoses, the history of organ transplant, the presence of HIV infection). Data are presented as means, standard deviation and range, and as proportions for categorical variables. A Wilcoxon rank sum test, Pearson's Chi‐squared test, and Fisher's exact test, when appropriate, were used to test differences between variables. The statistical analyses were conducted using the R software package version 4.0.2 (https://cran.rstudio.com) and *p* < 0.05 was considered statistically significant.

Approval of an ethics committee was not required as the research was retrospective and based on medical records only.

## 3 RESULTS

**Figure 2:**
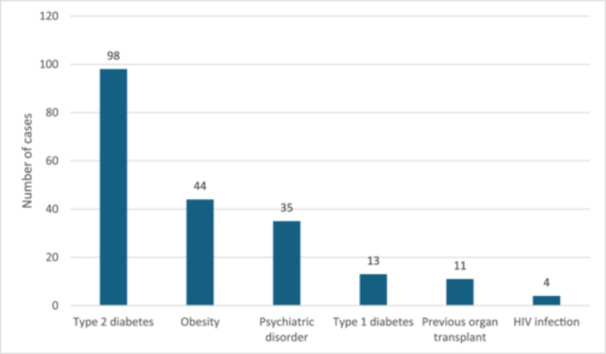
Comorbidities among dermatology outpatient clinic patients with a diagnosed fungal infection.

The data query returned 667 cases with the aforementioned codes. When unverified cases were excluded, there were a total of 573 cases with 734 superficial fungal infections included in this study. Most cases were males (*n *= 330; 57.6%) and the mean age of the patients was 58 years (Tables 1 and 2). The most common comorbidities were type 2 diabetes (17.1%) and obesity (7.7%) (Figure 2) and 16.8% of the cases were using immunosuppressive medication.

**Table 1:** Characteristics of fungal infections in males and females.

Characteristic	Male, *n* = 330	Female, *n* = 243	Overall, *n* = 573	*p* Value
Number of infections^a^				0.006^b^
1	233 (70.6)	195 (80.2)	428 (74.7)	
2	84 (25.5)	47 (19.3)	131 (22.9)	
3	11 (3.3)	1 (0.4)	12 (2.1)	
4	2 (0.6)	0 (0.0)	2 (0.3)	
Previous fungal infection				0.8^b^
No	70 (21.2)	44 (18.1)	114 (19.9)	
Yes, one	64 (19.4)	49 (20.2)	113 (19.7)	
Yes, more than one	13 (3.9)	8 (3.3)	21 (3.7)	
No information	183 (55.5)	142 (58.4)	325 (56.7)	
Age, years, mean (SD)	59 (16)	57 (18)	58 (17)	0.124^c^
Age group *n* (%)				0.054^b^
18–40 years	49 (14.8)	55 (22.6)	104 (18.2)	
41–60 years	106 (32.1)	74 (30.5)	180 (31.4)	
Over 61 years	175 (53.0)	114 (46.9)	289 (50.4)	
Fungal infection was the reason for referral^d^				0.786^b^
No	204 (61.8)	148 (60.9)	352 (61.4)	
Yes	124 (37.6)	92 (37.9)	216 (37.7)	
No information	2 (0.6)	3 (1.2)	5 (0.9)	

*Note*: Presented as *n* (%) unless other stated.

Abbreviation: SD, standard deviation.

^a^ Includes all cumulative fungal infections during the study period.

^b^ Pearson's Chi‐squared test; Fisher's exact test.

^c^ Wilcoxon rank sum test.

^d^ For the first visit to dermatology clinic due to the fungal infection.

**Table 2:** Characteristics of patients and diagnosed fungal infections based on anatomical site of infection.

	Tinea unguium of toenails (*n* = 275)	Tinea unguium of fingernails (*n* = 36)	Tinea pedis (*n* = 295)	Tinea corporis (*n* = 72)	Tinea capitis (*n* = 3)	Tinea cruris (*n *= 53)	Total (*n* = 734)
Sex							
Male	160 (58.2)	28 (77.8)	171 (58.0)	39 (54.2)	1 (33.3)	43 (81.1)	442 (60.2)
Female	115 (41.8)	8 (22.2)	124 (42.0)	33 (45.8)	2 (66.7)	10 (18.9)	292 (39.8)
Age, mean (SD)	57 (16)	63 (18)	58 (17)	62 (17)	77 (10)	56 (20)	58 (17)
Age group							
18‐40 years	46 (16.7)	5 (13.9)	54 (18.3)	9 (12.5)	0	14 (26.4)	128 (17.4)
41‐60 years	102 (37.1)	9 (25.0)	93 (31.5)	17 (23.6)	0	14 (26.4)	235 (32.0)
Over 61 years	127 (46.2)	22 (61.1)	148 (50.2)	46 (63.9)	3 (100)	25 (47.2)	371 (50.5)
Diagnostic method							
Fungal culture only	130 (47.3)	25 (69.4)	105 (35.6)	31 (43.1)	2 (66.7)	16 (30.2)	309 (42.1)
NAAT only	121 (44.0)	12 (33.3)	125 (42.4)	27 (37.5)	1 (33.3)	8 (15.1)	294 (40.1)
Fungal culture and NAAT	4 (1.5)	1 (2.8)	3 (1.0)	2 (2.8)	0 (0.0)	0 (0.0)	10 (1.4)
Clinical picture only	25 (9.1)	0 (0.0)	67 (22.7)	12 (16.7)	0 (0.0)	26 (49.1)	130 (17.7)
Fungal pathogen^a^							
*Trichophyton rubrum*	208 (83.2)	31 (83.8)	179 (77.8)	45 (62.5)	2 (77.5)	18 (75.0)	483 (80.1)
*Trichophyton interdigitale*	6 (2.4)	0 (0.0)	16 (7.0)	1 (1.4)	0 (0.0)	0 (0.0)	23 (3.8)
*Trichophyton mentagrophytes*	7 (2.8)	0 (0.0)	10 (4.3)	3 (4.2)	0 (0.0)	0 (0.0)	20 (3.3)
*Trichophyton tonsurans*	0 (0.0)	0 (0.0)	0 (0.0)	1 (1.4)	0 (0.0)	0 (0.0)	1 (0.2)
*Epidermophyton floccosum*	1 (0.4)	0 (0.0)	1 (0.4)	0 (0.0)	0 (0.0)	0 (0.0)	2 (0.3)
*Candida albicans*	0 (0.0)	1 (2.7)	1 (0.4)	0 (0.0)	0 (0.0)	3 (12.5)	5 (0.8)
Other pathogen^b^	8 (3.2)	4 (10.8)	3 (1.3)	1 (1.4)	0	1 (0.04)	17 (2.8)
Fungal infection was the reason for referral	99 (36.0)	17 (47.2)	102 (34.6)	33 (45.8)	2 (66.7)	32 (60.4)	285 (38.8)
The duration of symptoms before the diagnosis in the dermatology clinic^c^							
<1 month	2 (2.0)	0 (0.0)	9 (8.8)	3 (9.1)	0 (0.0)	3 (9.4)	17 (6.0)
1–2 months	6 (6.1)	1 (5.9)	5 (4.9)	6 (18.2)	0 (0.0)	4 (12.5)	22 (7.7)
3–6 months	15 (15.2)	3 (17.6)	13 (12.7)	3 (9.1)	0 (0.0)	4 (12.5)	38 (13.3)
>6 months	51 (51.5)	9 (52.9)	48 (47.1)	13 (39.4)	1 (50.0)	9 (28.1)	131 (46.0)
The treatment of the infection^d^							
Topical treatment only	50 (18.2)	5 (13.9)	139 (47.1)	29 (40.3)	0	32 (60.4)	
Oral antifungal therapy only	139 (50.5)	17 (47.2)	77 (26.1)	19 (26.4)	0	4 (7.5)	
Topical and oral therapy	46 (16.2)	10 (2.8)	67 (22.7)	22 (30.6)	3 (100.0)	17 (32.1)	
No treatment	24 (8.7)	2 (5.6)	3 (1.0)	0	0	0	

*Note*: Presented as *n* (%) unless other stated.

Abbreviations: NAAT, nucleic acid amplification test; SD, standard deviation.

^a^ Of those infections where fungal culture or NAAT was taken.

^b^ For example, *Cryptococcus yeast* and Fusarium sp.(tinea unguim); Candidaparapsilosis, Cryptococcus sp., Trichosporon (tinea pedis); *Candida parapsilosis* (tinea corporis).

^c^ Among those referred due to fungal infection (there may be some missing data).

^d^ Some patients did not want any treatment, in addition, there may be some missing data.

**Figure 3:**
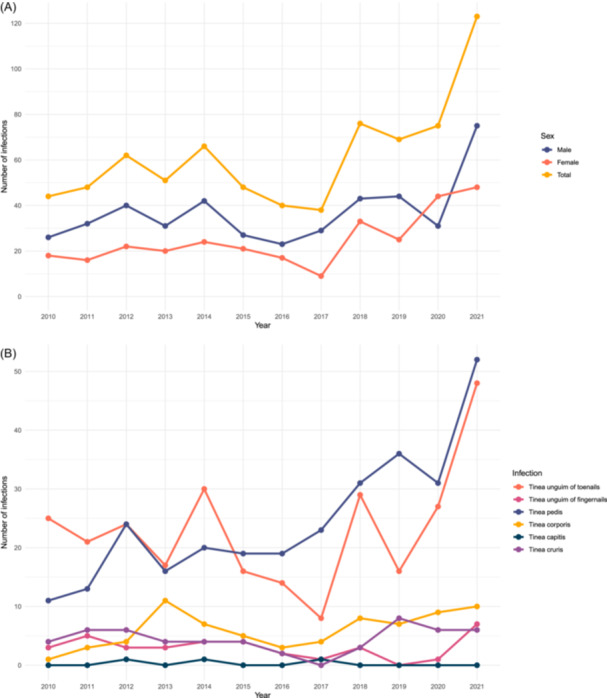
Number of diagnosed fungal infections between 2010 and 2021 by sex (A) and anatomical location (B).

From the year 2017 onward, there was an increase in the number of diagnosed fungal infections (Figure 3).

Diagnosed or suspected fungal infection was the reason for referral from primary care to a dermatologist in 216 patients (37.7%). The delay from the onset of symptoms to diagnosis in these patients (*n *= 216) was long in all tinea types; over one‐third to half of the cases had had symptoms for more than 6 months before the dermatology visit (Table 2).

All fungal infections except for tinea capitis were more common in males than in females. The most common fungal infection was tinea pedis (*n *= 295), seen in over half of the patients (51.5%), followed by tinea unguium (*n *= 275, 48.0%) (Table 2). Concomitant tinea pedis and tinea unguium of toenails were present in 108 (18.8%) of all patients; this was the most common combination of fungal infections. There were only three patients with tinea capitis (0.5%) in our study population. Most patients had one fungal infection (*n *= 428,74.7%). One‐fourth (25.3%) of the subjects had several cumulative fungal infections; males more often than females (*p *< 0.005). Nearly a fifth (19.7%, *n *= 113) of the patients had a previous fungal infection reported in their medical history and 21 patients (3.7%) had several superficial fungal infections. All fungal infections were more common in the oldest age group (Table 2).

During the study period, the predominant diagnostic method for fungal infections of the skin changed: NAAT‐based method became more prevalent than fungal culture, surpassing it in numbers in 2016 (Supporting Information S1: Figure S1). Other methods of diagnosis were rare (i.e., skin biopsy, data not shown).

Overall, the most commonly found dermatophyte was *T. rubrum*. For tinea unguium in fingernails, toenails and tinea pedis, it was found in 83.8%, 83.2%, and 77.8%, respectively, of the samples taken. The second most common pathogens causing tinea pedis were *Trichophyton interdigitale* (7.0%) and *Trichophyton mentagrophytes* (4.3%). All identified fungal pathogens and their anatomical locations are shown in Table 2 as are treatment alternatives in each fungal infection.

## 4 DISCUSSION

In this longitudinal study spanning more than a decade, there were 573 adult patients diagnosed with a superficial fungal infection in OUH in dermatology clinic with a male predominance. Regarding the age, any tinea was the most common among cases over 61 years. During the study time, the number of diagnosed fungal infections increased; the increase was seen especially in toenail onychomycosis and tinea pedis. According to this study, the superficial fungal skin infections rather commonly lead to dermatologist referral.

Skin‐related problems—including superficial fungal infections—are frequent reasons to visit a doctor in a primary care in Finland.^4^ Nevertheless, according to this study, a high number of patients are treated because of them in secondary care, too. Surprisingly, only one‐third of the patients of our study population were referred to the dermatology clinic because of a suspected dermatophyte infection and in turn, the majority had fungal infection as a secondary diagnosis detected during a dermatology visit for some other reason. Moreover, we demonstrated a long diagnostic delay in all fungal infections. This could be explained by the difficulty of recognizing and diagnosing a dermatophyte infection since other diseases may cause similar symptoms as fungal infections. For example, eczema can mimic tinea corporis, and dystrophic nails can look similar to nails in tinea unguium.^10^ In addition, fungal infections do not always cause prominent symptoms, which can make the diagnosis challenging both for physicians and patients itself.

In our study, there was a trend of increasing incidence of superficial fungal infections in secondary care. There is a lack of comparative studies performed in the same geographical area but our finding is in line with a previous study from Singapore which showed a higher prevalence in particular of onychomycosis and tinea pedis in recent years.^16^ However, the existing literature offers only limited data about the incidence of these diseases as studies focus more on the changing spectrum of dermatophytes in fungal infections.^3^, ^17^, ^18^, ^19^ Nevertheless, better diagnostic methods, such as increased use of a very sensitive dermatophyte NAAT assay, could explain our increasing incidence. In recent years, NAAT assay has been used in several primary care settings in NOHD area beside the OUH. In addition, fungal skin infections are associated with many somatic diseases such as diabetes,^20^ and as the number of diabetic patients is increasing,^21^ it is likely that this is also reflected in the number of fungal infections. In the present study, nearly 17% of the patients had diabetes as a comorbidity: this is slightly more than the prevalence of diabetes in the general Finnish population among those aged 55–64 years, as 11.3% of males and 6.5% of females had type 2 diabetes in a national health study.^22^, ^23^ Moreover, Finnish people are traveling more and more abroad, which may have effect to the increase of the superficial fungal infections. It has also become a trend to spend more leisure time in health clubs and in spas in which the risk for transmission of dermatophytes is higher. It is also possible—based on the present study—that there are difficulties in recognition of fungal skin infections in primary care and due to the uncertainty, they are referred to the secondary care.

Similarly to many other studies,^7^, ^8^, ^10^, ^15^, ^16^ we found that overall, fungal infections affect more males than females. This gender difference may result from many factors; the comorbidities associating with fungal infections are more prevalent in males than in females,^21^ and, for example, tinea cruris favors males due to anatomical factors. Moreover, females probably take more care of their skin, seek more readily help for their symptoms^24^ and have lower risk of occupational fungal disease (i.e., males wearing protective footwear more often).

The most common fungal infections in this study were tinea pedis and tinea unguium of the toes. Many patients were also diagnosed with both simultaneously. Similarly, in the two population‐based studies from Finland among adults and older persons, these fungal infections were the most common ones.^6^, ^7^, ^8^ Corresponding findings have been reported outside Finland as well: In the large pan‐European Achilles survey performed in 1997 and 1998 (*n* = 98.235), the prevalence of tinea pedis was over 50%, confirming that fungal skin infections are extremely common, and importantly, not just a cosmetic problem but a major risk factor for cellulitis, for example.^25^ In line with other studies,^7^, ^26^ in the present study, all fungal infections affected the oldest age group (61–96 years) more commonly compared to others. Tinea capitis was only found in very few adult patients in our study. This is not surprising, as tinea capitis mainly affects children^27^ due to the differences in biological factors of the hair between adults and children.^28^

*T. rubrum* was the most frequently appearing dermatophyte in all examined fungal infections in our study. Similarly, it is the most common dermatophyte globally, regardless of geographical location, and its role as a causative agent has been increasing worldwide.^3^ Other common dermatophytes in our study were *T. interdigitale* and *T. mentagrophytes*, both causing tinea pedis, tinea unguium of the toes, and tinea corporis. These findings align with other studies from Europe and the United States.^15^, ^17^ Interestingly, we found only one fingernail onychomycosis caused by *Candida albicans* (the majority, 86%, were caused by *T. rubrum*), which differs markedly from other studies.^16^, ^17^ It is of great importance to be aware of exact pathogens causing fungal infections since, for example, *T. indotineae* has caused difficult‐to‐treat dermatophytosis especially in India but also recently in Scandinavia.^12^, ^29^

Slight over half of the patients with tinea unguium of the toes were treated with oral antifungal therapy which is the most common treatment option in onychomycosis. However, even 18% of the patients had been treated only with topical treatment. Even though oral antifungals have a better success rate and shorter length of use time as their benefit, they indisputably have more serious side effects than topical treatments.^30^ These facts probably explain the surprisingly high number of patients treated with topicals only. The type of treatment in tinea corporis, instead, depends on the disease severity. In the present study, half of the patients with tinea corporis were treated with oral antifungals. Unfortunately, we could not analyze the outcomes of the treatment, since only very few patients with superficial fungal infection need a follow‐up visit in dermatology clinic and thus the outcomes cannot be retrospectively verified.

The main strength of this study is its comprehensive and large population and longitudinal study design. All patients were diagnosed by experienced dermatologists, and the vast majority of fungal diseases were verified by NAAT or fungal culture, confirming the diagnoses further. We admit that we also included the cases that were diagnosed by clinical picture since, for example, it is acceptable to treat fungal infection in toe webs without fungal sampling according to the Finnish Current Care Guidelines.^11^ Moreover, during the SARS‐CoV‐2 pandemic there was a period during which dermatophyte‐NAAT was unavailable, as all NAAT resources were targeted to COVID samples. However, in these cases the clinical photographs were further evaluated by experienced dermatologists (S. P. S. or L. H.). One specific strength of this study is that it did not focus only on the dermatophytes behind fungal infections but broadens the understanding by also analyzing the other characteristics of the study population and the incidence of fungal infections over a period spanning more than a decade. The study has some limitations: since the study population included only adults from an area with mainly Caucasian population, the study results may not be generalized to other populations. Moreover, since the study was performed in secondary care, the results are comparable only with similar study design. As this was a retrospective study, there was also some missing data not found in the EHR (i.e., in some cases, the duration of the symptoms) and due to the study design, we could not verify the correctness of the diagnosis.

In conclusion, the incidence of superficial fungal infections in adults treated in secondary care seems to be increasing. In our study, a markedly long delay from the onset of symptoms to the diagnosis in the dermatology clinic was demonstrated. Majority of the subjects had fungal infection as a secondary diagnosis detected during a dermatology visit. Thus, although the diagnostic methods used to recognize dermatophytes have improved, more knowledge about these diseases is needed to further improve the diagnostics. Recognizing and treating fungal infections without delay might hinder the increase of resistant pathogens and recalcitrant infections recently seen also in Europe.^31^ It is also wise to be aware of preventative methods of fungal infections such as good hygiene and the use of the protective shoes, that is, in general gyms to prevent the transmission of infection.

## AUTHOR CONTRIBUTIONS

**Teemu Mäntyniemi**: Investigation; writing—original draft. **Suvi Säntti**: Investigation; writing—original draft. **Eetu Kiviniemi**: Formal analysis; methodology; software. **Jari Jokelainen**: Formal analysis; software; methodology; visualization. **Laura Huilaja**: Conceptualization; data curation; project administration; supervision; writing—review and editing. **Suvi‐Päivikki Sinikumpu**: Conceptualization; data curation; project administration; supervision; writing—review and editing.

## CONFLICT OF INTEREST STATEMENT

The authors declare no conflict of interest.

## ETHICS STATEMENT

The approval of the medical director of OUH was received for the study.

## TRANSPARENCY STATEMENT

The lead author Suvi‐Päivikki Sinikumpu affirms that this manuscript is an honest, accurate, and transparent account of the study being reported; that no important aspects of the study have been omitted; and that any discrepancies from the study as planned (and, if relevant, registered) have been explained.

## Supporting information

Supporting information.

## Data Availability

Data requests can be submitted to hospital administrative authority.

## References

[1] Ameen M . Epidemiology of superficial fungal infections. Clin Dermatol. 2010;28:197‐201. 10.1016/j.clindermatol.2009.12.005 20347663

[2] Havlickova B , Czaika VA , Friedrich M . Epidemiological trends in skin mycoses worldwide. Mycoses. 2008;51:2‐15. 10.1111/j.1439-0507.2008.01606.x 18783559

[3] Hayette MP , Sacheli R . Dermatophytosis, trends in epidemiology and diagnostic approach. Curr Fungal Infect Rep. 2015;9:164‐179. 10.1007/s12281-015-0231-4

[4] Salava A , Oker‐Blom A , Remitz A . The spectrum of skin‐related conditions in primary care during 2015‐2019—A Finnish nationwide database study. Skin Health Dis. 2021;1(3):1‐9. 10.1002/SKI2.53 PMC9060089 35663141

[5] Martinez‐Rossi NM , Peres NTA , Bitencourt TA , Martins MP , Rossi A . State‐of‐the‐art dermatophyte infections: epidemiology aspects, pathophysiology, and resistance mechanisms. J Fungi (Basel). 2021;7(8):1‐17. 10.3390/JOF7080629 PMC8401872 34436168

[6] Faure‐Cognet Fricker‐Hidalgo H , Pelloux MT , Leccia OH . Superficial fungal infections in a French teaching hospital in Grenoble area: retrospective study on 5470 samples from 2001 to 2011. Mycopathologia. 2016;181:59‐66. 10.1007/s11046-015-9953-7 26452757

[7] Sinikumpu SP , Jokelainen J , Haarala AK , Keränen MH , Keinänen‐Kiukaanniemi S , Huilaja L . The high prevalence of skin diseases in adults aged 70 and older. J Am Geriatr Soc. 2020;68:2565‐2571. 32754902 10.1111/jgs.16706

[8] Sinikumpu SP , Huilaja L , Jokelainen J , et al. High prevalence of skin diseases and need for treatment in a middle‐aged population. A northern Finland birth cohort 1966 study. PLoS One. 2014;9(6):e99533. 24911008 10.1371/journal.pone.0099533 PMC4049840

[9] Sinikumpu SP , Auvinen J , Jokelainen J , et al. Abnormal skin in toe webs is a marker for abnormal glucose metabolism. A cross‐sectional survey among 1,849 adults in Finland. Sci Rep. 2017;7(1):9125. 10.1038/S41598-017-09354-3 28831117 PMC5567349

[10] Kovitwanichkanont T , Chong A . Superficial fungal infections. Aust J Gen Pract. 2019;48(10):706‐711. 10.31128/AJGP-05-19-4930 31569324

[11] Skin Infections . Current care guidelines. Working group set up by the Finnish Medical Society Duodecim and the Finnish Dermatol Society, Helsinki: *The Finnish Medical Society Duodecim* , 2017. www.kaypahoito.fiSuomalaisen

[12] Chowdhary A , Singh A , Kaur A , Khurana A . The emergence and worldwide spread of the species Trichophyton indotineae causing difficult‐to‐treat dermatophytosis: a new challenge in the management of dermatophytosis. PLoS Pathog. 2022;18(9):e1010795. 10.1371/JOURNAL.PPAT.1010795 36173977 PMC9521800

[13] Bao F , Fan Y , Sun L , et al. Comparison of fungal fluorescent staining and ITS rDNA PCR‐based sequencing with conventional methods for the diagnosis of onychomycosis. J Eur Acad Dermatol Venereol. 2018;32(6):1017‐1021. 10.1111/JDV.14843 29405481 PMC6001524

[14] Powell J , Porter E , Field S , O'Connell NH , Carty K , Dunne CP. Epidemiology of dermatomycoses and onychomycoses in Ireland (2001–2020): a single‐institution review. Mycoses. 2022;65(7):770‐779. 10.1111/MYC.13473 35598177 PMC9327510

[15] Svejgaard EL , Nilsson J . Onychomycosis in Denmark: prevalence of fungal nail infection in general practice. Mycoses. 2004;47:131‐135. 10.1111/j.1439-0507.2004.00968.x 15078429

[16] Tan HH . Superficial fungal infections seen at the National Skin Centre, Singapore. Nippon Ishinkin Gakkai Zasshi. 2005;46(2):77‐80. 10.3314/JJMM.46.77 15864251

[17] Foster KW , Ghannoum MA , Elewski BE . Epidemiologic surveillance of cutaneous fungal infection in the United States from 1999 to 2002. J Am Acad Dermatol. 2004;50:748‐752. 10.1016/S0190-9622(03)02117-0 15097959

[18] Gamage H , Sivanesan P , Hipler UC , Elsner P , Wiegand C . Superficial fungal infections in the department of dermatology, University Hospital Jena: a 7‐year retrospective study on 4556 samples from 2007 to 2013. Mycoses. 2020;63(6):558‐565. 10.1111/MYC.13077 32187409

[19] Khodadadi H , Zomorodian K , Nouraei H , et al. Prevalence of superficial‐cutaneous fungal infections in Shiraz, Iran: a five‐year retrospective study (2015‐2019). J Clin Lab Anal. 2021;35(7):1‐6. 10.1002/JCLA.23850 PMC8274978 34028857

[20] Cooke FJ . Infections in people with diabetes. Medicine. 2015;43:41‐43.

[21] Liu J , Ren ZH , Qiang H , et al. Trends in the incidence of diabetes mellitus: results from the Global Burden of Disease Study 2017 and implications for diabetes mellitus prevention. BMC Public Health. 2020;20:1415. 10.1186/s12889-020-09502-x 32943028 PMC7500018

[22] Finnish Institute for Health and Welfare .; 2021. https://thl.fi/en/web/thlfi-en/statistics

[23] Katja B , Liisa S , Laura L . 2013.Kansallinen FINRISKI 2012—Terveystutkimus—Osa I: Tutkimuksen Toteutus Ja Menetelmät.

[24] Rafuse J . Men's attitudes about seeking health care may put them at risk, conference told. Can Med Assoc J. 1993;149(3):329‐330. 8339180 PMC1485516

[25] Md Nor N , Inn Shih K , Jamil A , Mohd Nawi A . The risk factors of lower limb cellulitis: a case‐control study in a tertiary centre. Malays Fam Physician. 2020;15(1):23‐29. 32284801 PMC7136668

[26] Kim SH , Cho SH , Youn SK , et al. Epidemiological characterization of skin fungal infections between the years 2006 and 2010 in korea. Osong Public Health Res Perspect. 2015;6(6):341‐345. 10.1016/j.phrp.2015.10.012 26835243 PMC4700767

[27] Gupta AK , Friedlander SF , Simkovich AJ . Tinea capitis: an update. Pediatr Dermatol. 2022;39(2):167‐172. 10.1111/PDE.14925 35075666

[28] Hay RJ . Tinea capitis: current status. Mycopathologia. 2017;182:87‐93. 10.1007/s11046-016-0058-8 27599708 PMC5283510

[29] Astvad KMT , Hare RK , Jørgensen KM , Saunte DML , Thomsen PK , Arendrup MC . Increasing terbinafine resistance in Danish trichophyton isolates 2019–2020. J Fungi (Basel). 2022;8(2):150. 10.3390/JOF8020150 35205904 PMC8879722

[30] Gupta AK , Stec N , Summerbell RC , et al. Onychomycosis: a review. J Eur Acad Dermatol Venereol. 2020;34:1972‐1990. 32239567 10.1111/jdv.16394

[31] Abdolrasouli A , Hay RJ . Antifungal‐resistant Trichophyton indotineae: transmission is occurring outside previously identified endemic areas—are we prepared? BJD. 2024;191:1‐2. 10.1093/bjd/ljae140 38593243

