# Supplementary figures and images for: Superficial fungal infections in adults in Northern Finland between 2010 and 2021: A register‐based study
Source: Health Sci Rep. 2024 Oct 10;7(10):e70138. doi: 10.1002/hsr2.70138 (PMC11466830; doi:10.1002/hsr2.70138)

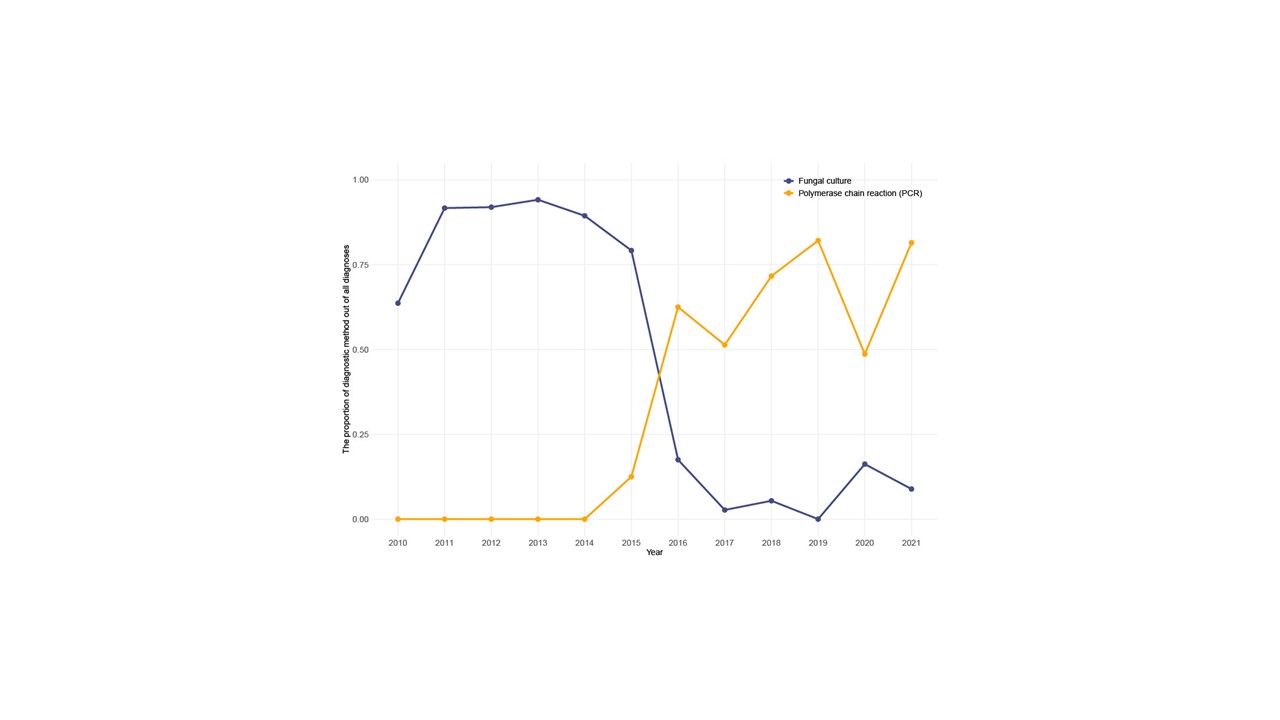

Supplement: Supplementary file 1 — Supporting information. [file HSR2-7-e70138-s001.jpg]
